# Beyond survival: identifying what matters to survivors of critical illness

**DOI:** 10.1186/s13054-021-03565-x

**Published:** 2021-04-06

**Authors:** Somnath Bose, Benjamin Hoenig, Maria Karamourtopoulos, Valerie Banner-Goodspeed, Samuel Brown

**Affiliations:** 1 Department of Anesthesia, Critical Care and Pain Medicine, Harvard Medical School, Beth Israel Deaconess Medical Center, One Deaconess Road, Rosenberg 470, Boston, MA 02215 USA; 2 Department of Anesthesia, Critical Care and Pain Medicine, Center for Anesthesia Research Excellence (CARE), Beth Israel Deaconess Medical Center, Boston, MA 02215 USA; 3 Present Address: Albany Medical College, Albany, NY 12208 USA; 4 Center for Humanizing Critical Care and Pulmonary Critical Care Medicine, Intermountain Medical Center, Murray, UT 84107 USA; 5 Pulmonary and Critical Care Medicine, University of Utah, Salt Lake City, UT 84132 USA

Critical care has made great strides in the last 50 years. Advances have led to significant reduction in hospital mortality despite an increase in severity of illness [1]. Though encouraging, survivors of critical illness often endure long-term sequelae across multiple domains which can be debilitating and life changing. These *new or worsening* impairments often persist years beyond index hospitalization. The impact of these impairments on patients, families, and healthcare systems cannot be underestimated [2]. Although majority of patients are grateful to have survived even in the face of significant disability, some may regret having survived because of new or worsened disabilities.

Research in critical care has focused on mortality as the primary outcome of interest. The choice of a *binary* outcome: mortality being the focus of most trials is rational and intuitive. The importance of survival remains beyond debate, although intensive care unit stays for some represent a temporary stop in the natural dying process. Realistically, majority of survivors have expectations *beyond* survival [3]. Therefore, the current practice of assessing interventions primarily on the basis of how effectively they influence mortality *only* deserves scrutiny.

## Non-mortality endpoints in clinical trials

Surviving an ICU stay matters overwhelmingly. Conditional on survival, there are multiple other facets which matter substantially [4]. A study among survivors demonstrated that some perceived the burden of survivorship as “*worse than death*” [5]. Although a minority hold this view, most patients and caregivers are accepting of *tragic trade-offs* associated with survival. From a practical standpoint, “affective forecasting” in the setting of new disabilities remains challenging and is unpredictably influenced by patients’ resilience and adaptation [6]. This underpins the importance of looking *beyond* mortality and derived measures. So how do we identify areas which deserve prioritization in terms of developing and testing treatment strategies based on patients’ perspectives?

Current critical care literature is rife with trials which have not demonstrated meaningful mortality reduction, while this could be attributed to trials being under-powered or study populations being heterogeneous, the trend remains disappointing. A systematic review of 212 trials provided no conclusive evidence of any single pharmacological intervention translating to mortality benefit [7]. Interestingly, another meta-analysis concluded that patient-important outcomes other than mortality were *seldom* primary outcomes [8] and commonly used surrogates *did not directly* matter to patients [9]. With this background, our current framework affords a limited insight into the impact of various interventions in this growing population. These issues are being increasingly recognized, culminating in calls for more efficient, patient-centered research [10].

Substantial work has been done in this area leading to development of core-outcome sets and validated measures to encourage standardized reporting and comparability between trials [11]. Although work on this topic engaged patients and caregivers, overall representation of such groups has been low [12]. Patients who do not survive for long are naturally excluded. For survivors, a single interview far removed from index episode introduces biases and is incapable of tracking “response shifts.” Our current understanding therefore remains primarily reflective of the perception of stakeholders other than patients or care-givers [12].

The key question remains: What matters *most* to survivors of critical illness and their caregivers and how could these priority areas be identified reliably?

Recovery is multidimensional with its extent varying across multiple domains, such as cognitive, physical, mental health, return to work or residence, and therefore identification of order of prioritization is a key initial step.

**Fig. 1:**
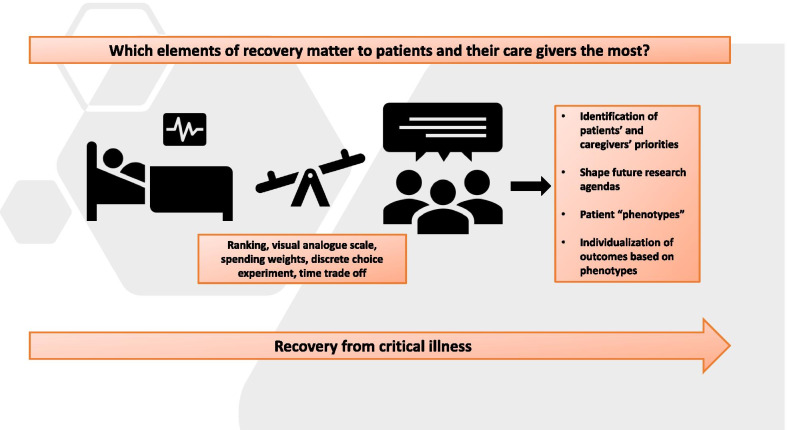
Identifying what matters to survivors of critical illness and their caregivers

We propose a framework to identify a hierarchical ranking of domains of recovery in the order of perceived importance by survivors (and caregivers) and following them over 3 and 6 months to assess their stability or shift following discharge (Fig. 1). For example, allowing survivors and caregivers of acute respiratory failure to assess trade-offs and rank recovery domains identified in prior Delphi work would help establish their perception of optimal recovery and ascertain relative importance of the components. The potential benefits of this approach include:

- Direct identification of patients’ and caregivers’ priorities which could be used to shape future research agendas.
- Preliminary identification of “patient phenotypes” based on their order of preference. For example, patients with certain baseline characteristics may favor cognitive recovery over physical function and vice versa.
- Individualization of endpoints based on “patient phenotypes.” The approach of individualized endpoints within the framework of trials is a viable strategy [13, 14]. In addition to its patient-centeredness this has been hypothesized to increase overall power by reducing the signal/noise ratio [13]. Arguably this framework may not necessarily fit into the framework of biological interventions but represents a viable option for testing supportive or rehabilitative strategies.

Eliciting a hierarchy of priorities relies on patients considering trade-offs between various aspects of recovery. Multiple approaches, some adopted from market research strategies could be applied to elicit such trade-offs. These methods range from using simple visual analogue scales, ranking, spending weights to more sophisticated approaches such as discrete choice experiments, standard gamble or time trade-off [15]. Eliciting responses from survivors could be difficult in the setting of new or worsened cognitive impairments. Similarly, when forced to *hypothetically* rank, survivors may find it challenging to distinguish between domains with overlap (for example physical function and return to work) or discriminate between major domains (such as cognition and physical function). It is conceivable that preferences elicited may be influenced by patient’s baseline functional status and socioeconomic considerations which by themselves may be informative. Finally, since predictive models are not necessarily accurate in predicting trajectories of individual patients following critical illness, these phenotypes identified may not be useful for advance care planning.

In conclusion, critical care research should emulate the paradigm of patient and family-centered care. Elucidation of priorities from survivors and caregivers represents a pragmatic approach towards designing more patient-centered trials. Understanding priorities should be a crucial factor in prioritizing future research agendas. Once identified, these constructs could be added to trial endpoints to ascertain how they interface with proposed interventions. Further, new support interventions could be developed drawing from the perspective of survivors and their caregivers. Broader impact of our interventions should be eventually measured vis-à-vis patient and caregivers’ preferences.

## Data Availability

Not applicable.
